# The spleen microbiota of small wild mammals reveals distinct patterns with tick-borne bacteria

**DOI:** 10.1371/journal.pntd.0006499

**Published:** 2018-07-05

**Authors:** Yan Ge, Guangpu Guo, Baoxue Ge, Hongmei Yin, Hong Yin

**Affiliations:** 1 Department of Immunology and Pathogen Biology, Tongji University School of Medicine, Shanghai, China; 2 Departement of Biotechnology, College of Life Sciences and Technology, Tongji University, Shanghai, China; 3 The State Key Laboratory of Veterinary Etiological Biology, Lanzhou Veterinary Research Institute, Chinese Academy of Agricultural Sciences, Gansu, China; Baylor College of Medicine, UNITED STATES

## Abstract

**Background:**

Wild mammals serve as reservoirs for a variety of microbes and play an important role in the enzootic cycles of these microbes. Some of them are vector-borne bacteria in the genera *Anaplasma*, *Ehrlichia* and *Rickettsia* of the order Rickettsiales, which can cause febrile illnesses in human beings as well as animals. *Anaplasma* spp., *Ehrlichia* spp. and many spotted fever group (SFG) *Rickettsia* spp. are transmitted to mammalian hosts by tick vectors during blood meals. As a powerful sequencing method, the next generation sequencing can reveal the complexity of bacterial communities in humans and animals. Compared with limited studies on blood microbiota, however, much fewer studies have been carried out on spleen microbiota, which is very scarce in wild mammals. Chongming Island is the third biggest island in China. It was unclear whether there were any vector-borne bacteria in Chongming Island. In the present study, we explored the bacterial microbiota in the spleens of wild mice and shrews from the rural areas of Chongming Island and investigated the prevalence of vector-borne bacteria.

**Methodology/Principal findings:**

Genomic DNAs were extracted from the spleen samples of 35 mice and shrews. The 16S rDNA V3-V4 regions of the DNA extracts were amplified by PCR and subjected to the 16S rDNA-targeted metagenomic sequencing on an Illumina MiSeq platform. All the 35 spleen samples obtained data with sufficient coverage (99.7–99.9%) for analysis. More than 1,300,000 sequences were obtained after quality control and classified into a total of 1,967 operational taxonomic units (OTUs) clustered at 97% similarity. The two most abundant bacterial phyla were Firmicutes and Proteobacteria according to the analysis of rarefied sequences. Among the bacterial communities detected in this study, *Anaplasma*, *Rickettsia* and *Coxiella* were adjacently clustered by hierarchical analysis. Significant differences in many bacterial features between *Anaplasma*-positive and *Anaplasma*-negative samples were identified by LEfSe analysis and Wilcoxon rank-sum test, suggesting that the *Anaplasma*-infection of small wild mammals was associated with a specific pattern of spleen microbiota.

**Conclusions/Significance:**

Our study has comprehensively characterized the complex bacterial profiles in the spleens of wild mice and shrews from Chongming Island, Shanghai city. This work has revealed distinct spleen bacterial communities associated with tick-borne bacteria in wild animals. The detection of tick-borne bacteria highlights the risk of contracting pathogens with public health importance upon tick-exposure in the studied areas.

## Author summary

In this study, the 16S rDNA-targeted metagenomic sequencing was used to determine the bacterial community and diversity in the spleens of small wild mammals from China. The 16S rDNAs were amplified from the spleen genomic DNAs of 35 small wild mice and shrews and sequenced by Illumina MiSeq technology. More than 1,300,000 sequences were obtained after quality control and classified into a total of 1,967 operational taxonomic units (OTUs) clustered at 97% similarity. The two most abundant bacterial phyla were Firmicutes and Proteobacteria according to the analysis of rarefied sequences. Within the bacterial communities detected in this study, vector-borne bacteria, *Anaplasma*, *Rickettsia* and *Coxiella*, were adjacently clustered by hierarchical analysis. Significant differences in many bacterial features between *Anaplasma*-positive and *Anaplasma*-negative samples were observed, suggesting that the infection of small wild mammals with *Anaplasma* is associated with a distinct pattern of spleen microbiota. This study has revealed the complex bacterial profiles in the spleens of wild mice and shrews. The detection of vector-borne bacteria highlights the role of wild mice and shrews as animal reservoirs with potential public health importance in the studied areas.

## Introduction

Wild mammals serve as reservoirs for a variety of microbes and play an important role in the enzootic cycles of these microbes. Some of them are vector-borne bacteria in the genera *Anaplasma*, *Ehrlichia* and *Rickettsia* of the order Rickettsiales. *Anaplasma* spp., *Ehrlichia* spp. and many spotted fever group (SFG) *Rickettsia* spp. are transmitted to mammalian hosts by tick vectors. They are obligate intracellular bacteria, and their main target cells are white blood cells, erythrocytes, platelets and/or vascular endothelia [1–3]. These bacteria have evolved adapted strategies to evade and/or suppress host protective immune responses and can cause febrile illnesses in animals and/or humans [1–3]. They have been gradually recognized as emerging pathogens of public health importance around the world [3–7]. The prevalence of these tick-borne bacteria has been increasingly reported in China. For instance, *Anplasma phagocytophilum*, *Anaplasma bovis*, *Anaplasma ovis*, *Anaplasma central*, *Anaplasma marginale*, *Anaplasma platys*, *Anaplasma capra*, *Ehrlichia chaffeensis*, *Ehrlichia canis*, *Candidatus Neoehrlichia mikurensis*, *Rickettsia heilongjiangiensis*, *Rickettsia sibirica*, *Rickettsia raoultii* and *Rickettsia conorii* have been detected in ticks, animals or humans in many provinces of China [4,8–19]. However, the existence of these bacteria in Shanghai city, China is still unknown.

Mammals are ecosystems that are inhabited by niche-specific microbiota including bacteria, viruses and fungi etc. The commensal microbiota plays essential roles in the development of immune system, modulation of metabolism and maintenance of health [20]. The perturbation of symbiotic microbiota has been shown to be associated with various diseases such as infection, immunological disorders, metabolic diseases and cancer etc. [20–22]. It had been thought that the circulatory system was sterile in healthy organisms, and that bacteria were present in the circulation only due to sepsis. Nevertheless, the presence of bacteria in the blood of healthy humans began to be documented several decades ago [23,24]. With the advance in sequencing technology, blood microbiota has been gradually uncovered in healthy organisms in the past decade [25,26].

The spleen, a peripheral lymphoid organ in vertebrates, acts as a blood filter. It plays an important role in the modulation of immune responses and hematopoiesis [27]. The spleen can be infected by the tick-borne bacteria from the order Rickettsiales [28–30]. During the establishment of intracellular infection in the spleen, these bacteria may have impacts on their host cells and alter the spleen niche. Therefore, we hypothesize that the changed spleen niche due to the infection with tick-borne bacteria would lead to the formation of specific spleen microbiota. As important reservoirs for the tick-borne bacteria, mice also serve as model animals for human infection. The present study explores the spleen microbiota in wild mice and shrews from Chongming Island, Shanghai city, China. The blood microbiota of wild mice from Israel and the spleen microbiota of wild voles from France have been recently reported, respectively, [31,32]. Compared with limited studies on blood microbiota, however, much fewer studies have been carried out on spleen microbiota, which is very scarce in wild mammals.

Chongming Island is the third biggest island in China. Its major part belongs to an administrative county of Shanghai city. It locates at the mouth of the Yangtze River. There has been no description on tick species present in Chongming Island yet. *Rhipicephalus sanguineus* and *Haemaphysalis longicornis*, however, have been reported to be the ticks infesting on pet dogs in other areas of Shanghai [33]. It is unclear whether there are any tick-borne bacteria in Chongming Island. The aim of the present study is to explore the bacterial microbiota in the spleens of wild animals from the rural areas of Chongming Island and investigate the presence of tick-borne bacteria.

## Methods

### Ethics statements

Animals were handled in accordance with National Guidelines for Ethic Review of Laboratory Animal Welfare. Animal treatment protocols were approved by the institutional animal ethics committee (the Animal Ethics Committee of Tongji University School of Medicine, Shanghai, China).

### Animal trapping

The Chongming Island has a humid subtropical monsoonal climate and a woodland habitat, which is suitable for tick infestation. Chongming Island has an area of around 1267 km^2^. The mouse collection sites mainly covered the middle and west parts of Chongming Island where there was more green coverage. Spring-loaded bar mousetraps with bait were used to trap mice. Traps were strategically placed in the environment such as crop fields, residential houses, bank of rivers and forests where wild mice were seen or expected living or traveling. These traps were set in the evening and checked in the next morning. The latitude and longitude of locations where mice and shrews were trapped were recorded through the Global Positioning System (GPS).

### Sample collection

Trapped live animals were transported in cardboard containers to the institutional Animal Biosafety Laboratory where they were euthanized by CO_2_. Necropsies were conducted after euthanasia. The spleen samples of trapped animals were collected and stored at –80°C.

### DNA extraction and PCR amplification

Genomic DNAs were extracted from the spleen samples using the E.Z.N.A. tissue DNA extraction kit (Omega Bio-tek, Norcross, GA, US) according to the manufacturer’s protocol. The quality and quantity of extracted DNAs were examined by 1% agarose gel electrophoresis and NanoDrop 2000 spectrophotometer (Thermo Scientific, MA, US). The V3-V4 regions of the 16S rDNA were amplified by PCR in a thermal cycler GeneAmp 9700 (Applied Biosystems Inc, Foster City, CA, US). The PCR condition was 95°C for 3 min, followed by 30 cycles at 95°C for 30 s, 55°C for 30 s, and 72°C for 45 s and a final extension at 72°C for 10 min. The primers used were 338F (5’-barcode-ACTCCTACGGGAGGCAGCAG-3’) and 806R (5’-GGACTACHVGGGTWTCTAAT-3’). The barcode is an eight-base sequence unique to each sample. The PCR reactions were performed in triplicate in 20 μL mixture containing 2 μL of 10 × PCR Buffer, 2 μL of 2.5 mM dNTPs, 0.8 μL of each primer (5 μM), 0.2 μL of rTaq DNA Polymerase (TaKaRa Bio, Dalian, China), and 10 ng of template DNA.

### Illumina MiSeq sequencing

The PCR amplicons were extracted from 2% agarose gels and purified using the AxyPrep DNA gel extraction kit (Axygen Biosciences, Union City, CA, US) according to the manufacturer’s instructions. After being quantified using QuantiFluor-ST (Promega, Madison, WI, US), the purified DNAs were pooled in equimolar and paired-end sequenced (2 × 300) on an Illumina MiSeq platform according to the standard protocols (Majorbio, Shanghai, China). The raw reads were deposited into the NCBI Sequence Read Archive (SRA) database (Accession Number: SRP118742).

### Processing of sequencing data

Raw fastq files were demultiplexed and quality-filtered using QIIME (version 1.9.1). The following criteria were met: (i) The 300 bp reads were truncated at any site with an average quality score < 20 over a 50 bp sliding window, discarding the truncated reads shorter than 50 bps. (ii) Exact barcode matching; maximal 2 nucleotide mismatches in primer matching. (iii) Only sequences overlapping longer than 10 bps were assembled according to their overlapped sequence. Reads containing ambiguous characters were removed. Reads that could not be assembled were discarded.

Operational taxonomic units (OTUs) were clustered with 97% similarity cutoff using UPARSE (version 7.1 http://drive5.com/uparse/) and chimeric sequences were identified and removed using UCHIME. The taxonomy of each 16S rRNA gene sequence was analyzed by RDP Classifier (http://rdp.cme.msu.edu/) against the SILVA (SSU123) 16S rRNA database using confidence threshold of 70% as previously described [34].

## Results

### Sequencing of samples

The small wild mammals trapped in the present study included mice and shrews. Thirty five samples submitted for 16S rDNA-targeted metagenomic sequencing were listed in Table 1. Except the four samples CS6, CS7, CS34 and CS88 from shrews, all the other 31 samples were from mice including *Apodemus agrarius*, *Mus musculus* and *Rattus flavipectus*. Shrews belong to the order Eulipotyphla.

**Table 1:** The detection of vector-borne bacteria, *Anaplasma*, *Ehrlichia*, *Rickettsia*, *Coxiella* and *Bartonella*, in the spleen samples of 35 small wild mammals from Chongming Island, Shanghai city.

**Samples**	**Animals**				***Anaplasma***		***Ehrlichia***	***Rickettsia***	***Coxiella***	***Bartonella***
	species sex environment (site) location				*A*. *ovis*	*A*. *phago*				
CS5	*Mus musculus*	M	R (JZ)	31°42.100 N 121°21.688 E						
CS6	shrew	M	F (JZ)	31°41.964 N 121°21.637 E	**+**			**+**	**+**	
CS7	shrew	F	F (JZ)	31°41.964 N 121°21.637 E				**+**		**+**
CS15	*Apodemus agrarius*	M	A (JZ)	31°42.192 N 121°21.758 E						
CS16	*Apodemus agrarius*	F	A (JZ)	31°42.192 N 121°21.758 E						
CS18	*Apodemus agrarius*	M	A (JZ)	31°42.192 N 121°21.758 E	**+**	**+**	**+**	**+**	**+**	**+**
CS19	*Apodemus agrarius*	F	A (JZ)	31°42.038 N 121°21.681 E				**+**		**+**
CS21	*Mus musculus*	M	A (JZ)	31°42.100 N 121°21.688 E						
CS30	*Rattus flavipectus*	F	R (MZ)	31°43.740 N 121°15.386 E				**+**		
CS34	shrew	F	R (MZ)	31°43.740 N 121°15.386 E				**+**		**+**
CS37	*Apodemus agrarius*	M	F (BH)	31°39.993 N 121°38.080 E	**+**	**+**		**+**	**+**	
CS38	*Apodemus agrarius*	M	F (BH)	31°39.993 N 121°38.080 E						**+**
CS39	*Apodemus agrarius*	F	F (BH)	31°39.993 N 121°38.080 E	**+**	**+**		**+**	**+**	
CS40	*Apodemus agrarius*	M	F (BH)	31°39.993 N 121°38.080 E						**+**
CS47	*Apodemus agrarius*	M	LB (BH)	31°39.856 N 121°38.216 E				**+**		
CS51	*Apodemus agrarius*	F	A (HX)	31°45.971 N 121°13.178 E				**+**		**+**
CS55	*Apodemus agrarius*	F	A (HX)	31°45.971 N 121°13.178 E	**+**	**+**	**+**	**+**	**+**	**+**
CS56	*Apodemus agrarius*	F	A (HX)	31°45.971 N 121°13.178 E				**+**		**+**
CS57	*Apodemus agrarius*	M	A (HX)	31°45.971 N 121°13.178 E	**+**		**+**	**+**	**+**	**+**
CS62	*Apodemus agrarius*	M	A (BH)	31°39.993 N 121°38.080 E	**+**	**+**		**+**	**+**	
CS63	*Apodemus agrarius*	F	A (BH)	31°39.993 N 121°38.080 E						**+**
CS65	*Apodemus agrarius*	M	A (BH)	31°39.993 N 121°38.080 E						
CS70	*Apodemus agrarius*	F	F (QW)	31°43.300 N 121°29.268 E						
CS72	*Apodemus agrarius*	M	A(QW)	31°43.300 N 121°29.268 E						
CS84	*Apodemus agrarius*	F	F (DP)	31°40.334 N 121°28.540 E						
CS85	*Apodemus agrarius*	F	F (DP)	31°40.334 N 121°28.540 E						**+**
CS88	shrew	M	RB (DP)	31°40.204 N 121°28.922 E				**+**		
CS90	*Apodemus agrarius*	M	F (DP)	31°40.334 N 121°28.540 E	**+**		**+**	**+**	**+**	**+**
CS91	*Apodemus agrarius*	F	F (DP)	31°40.334 N 121°28.540 E				**+**		
CS92	*Apodemus agrarius*	F	F (DP)	31°40.334 N 121°28.540 E	**+**		**+**	**+**	**+**	
CS97	*Apodemus agrarius*	M	F (DP)	31°40.334 N 121°28.540 E	**+**	**+**	**+**	**+**	**+**	**+**
CS98	*Apodemus agrarius*	M	F (DP)	31°40.334 N 121°28.540 E				**+**		**+**
CS99	*Apodemus agrarius*	M	F (DP)	31°40.334 N 121°28.540 E	**+**	**+**	**+**	**+**	**+**	**+**
CS110	*Mus musculus*	F	R (SX)	31°31.696 N 121°39.017 E						
CS118	*Apodemus agrarius*	F	R (DJ)	31°38.202 N 121°25.638 E						
Prevalence % (number of positive samples/number of tested samples)					**31.4% (11 /35)**	**20% (7/35)**	**20% (7/35)**	**60% (21/35)**	**31.4% (11/35)**	**45.7% (16/35)**

*A*. *phago*: *A*. *phagocytophilum*. Abbreviated environmental types: A, agricultural area; F, forest; LB, lake bank; R, residential area; RB, river bank near residential area. The latitudes and longitudes of locations where animals were trapped were provided.

In this study, all the 35 spleen samples obtained data with sufficient coverage (99.7–99.9%) for analysis. A total of 1,323,308 16S rRNA gene sequences with a read length of 469 bps were identified with an average of 37,808 reads per sample. And a total of 1,967 OTUs were clustered at 97% similarity across all samples. The number of OTUs per sample ranged from 127–528. The inverse Simpson’s diversity indices were from 0.072 to 0.6551, which indicated a broad variation in the bacterial diversity between samples. Rarefaction (to 17,808) resulted in 85–520 OTUs per sample.

### OTU abundance

**Fig 1:**
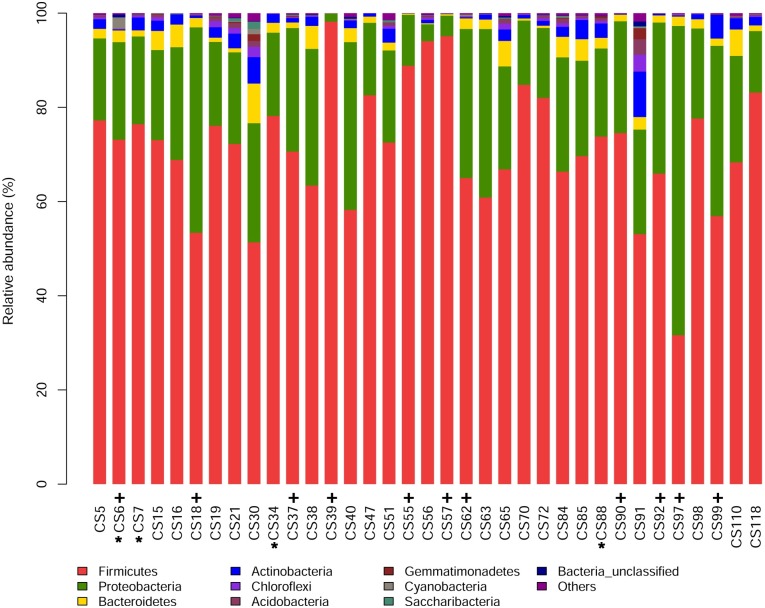
Spleen microbiota compositions at phylum level. Color-coded bar plot showing the proportion of different bacterial phyla present in 35 mouse and shrew samples from Chongming Island, Shanghai city. The 4 shrew samples were marked with *, and the left 31 samples were mice; Samples marked with + were *Anaplasma*-positive.

**Fig 2:**
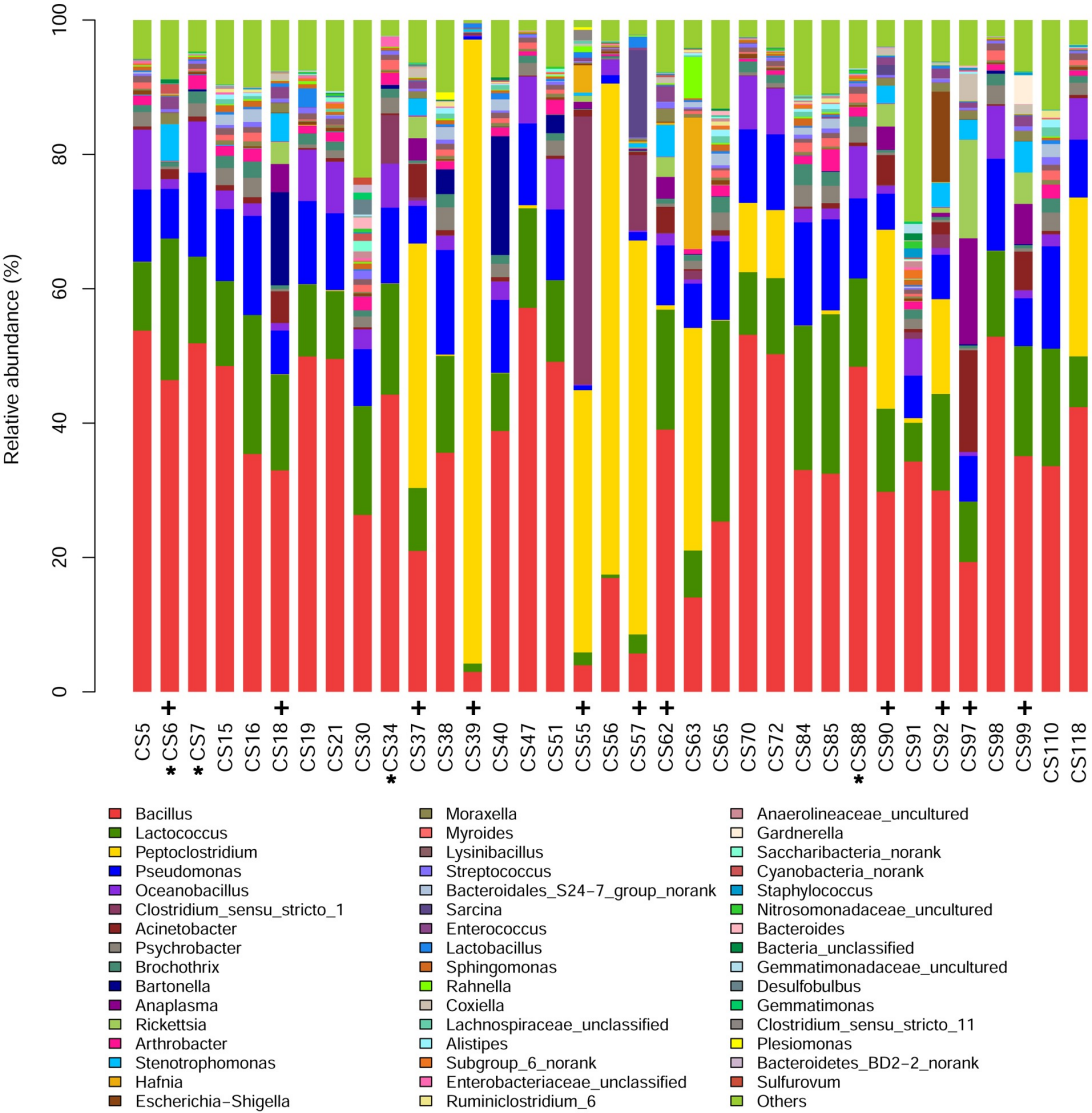
Spleen microbiota compositions at genus level. Color-coded bar plot showing the proportion of different bacterial genera present in 35 mouse and shrew samples from Chongming Island, Shanghai city. The 4 shrew samples were marked with *, and the left 31 samples were mice; Samples marked with + were *Anaplasma*-positive. Taxa with <1% abundance were listed as others.

Firmicutes was the most abundant phylum, and Proteobacteria was the second among the total taxa of 35 samples tested in this study except that the scenario for sample CS97 was inverse (Fig 1). Firmicutes and Proteobacteria had mean abundances of 71.53% (SD 13.48%) and 22.45% (SD 11.85%), respectively, in the total taxa of 35 samples (Fig 1). The sum of their mean abundances accounted for greater than 90% of the total taxa. The next three following bacterial phyla were Bacteroidetes, Actinobacteria and Choloriflexi with a mean abundance of 2.33% (SD 1.88%), 1.91% (SD 1.94%) and 0.47% (SD 0.73%), respectively (Fig 1). At genus level, there were 11 major bacterial taxa with a mean abundance greater than 1%, which included *Bacillus*, *Lactococcus*, *Peptoclostridium*, *Pseudomonas*, *Oceanobacillus*, *Clostridium_sensu_stricto_1*, *Acinetobacter*, *Psychrobacter*, *Brochothrix*, *Bartonella* and *Anaplasma* (Fig 2). In some samples e.g., CS39, CS55, CS56, CS57 and CS63, the relative abundance of *Peptoclostridium* was exceptionally high, whereas the relative abundances of *Bacillus* and *Lactococcus* were quite low.

### Vector-borne bacteria

The prevalence of vector-borne bacteria in the tested samples was summarized in Table 1. *Anaplasma* (*Anaplasma ovis* and/or *Anaplasma phagocytophilum*), *Ehrlichia*, *Rickettsia*, *Coxiella* and *Bartonella* were detected in 11, 7, 21, 11 and 16 of 35 (31.43%, 20%, 60%, 31.43% and 45.7%) samples, respectively. *Anaplasma* detected in the present study included two species, *A*. *ovis* and *A*. *phagocytophilum*. The relative abundance of *A*. *ovis* was much higher than that of *A*. *phagocytophilum* (S1 Table). *Ehrlichia* detected in the present study was much less abundant than *Anaplasma*, *Rickettsia*, *Coxiella* or *Bartonella* (S1 Table). *Coxiella* detected in the present study consisted of only one member, *Coxiella*_endosymbiont_of_*Rhipicephalus*_*turanicus* (S1 Table). All *Anaplasma*-positive samples were co-infected with *Coxiella* and vice versa. All *Ehrlichia*-positive samples were co-infected with *Anaplasma* and *Coxiella*, and all *Anaplasma*/*Coxiella*-positive samples were co-infected with *Rickettsia* but not *Bartonella*. *Anaplasma* (*A*. *ovis* and *A*. *phagocytophilum*), *Ehrlichia*, *Rickettsia*, *Coxiella* and *Bartonella* were detected in both the male and female animals. And they were all detected in the mouse samples. Except *A*. *phagocytophilum* and *Ehrlichia*, *A*. *ovis*, *Rickettsia*, *Coxiella* and *Bartonella* were detected in the shrew samples.

**Fig 3:**
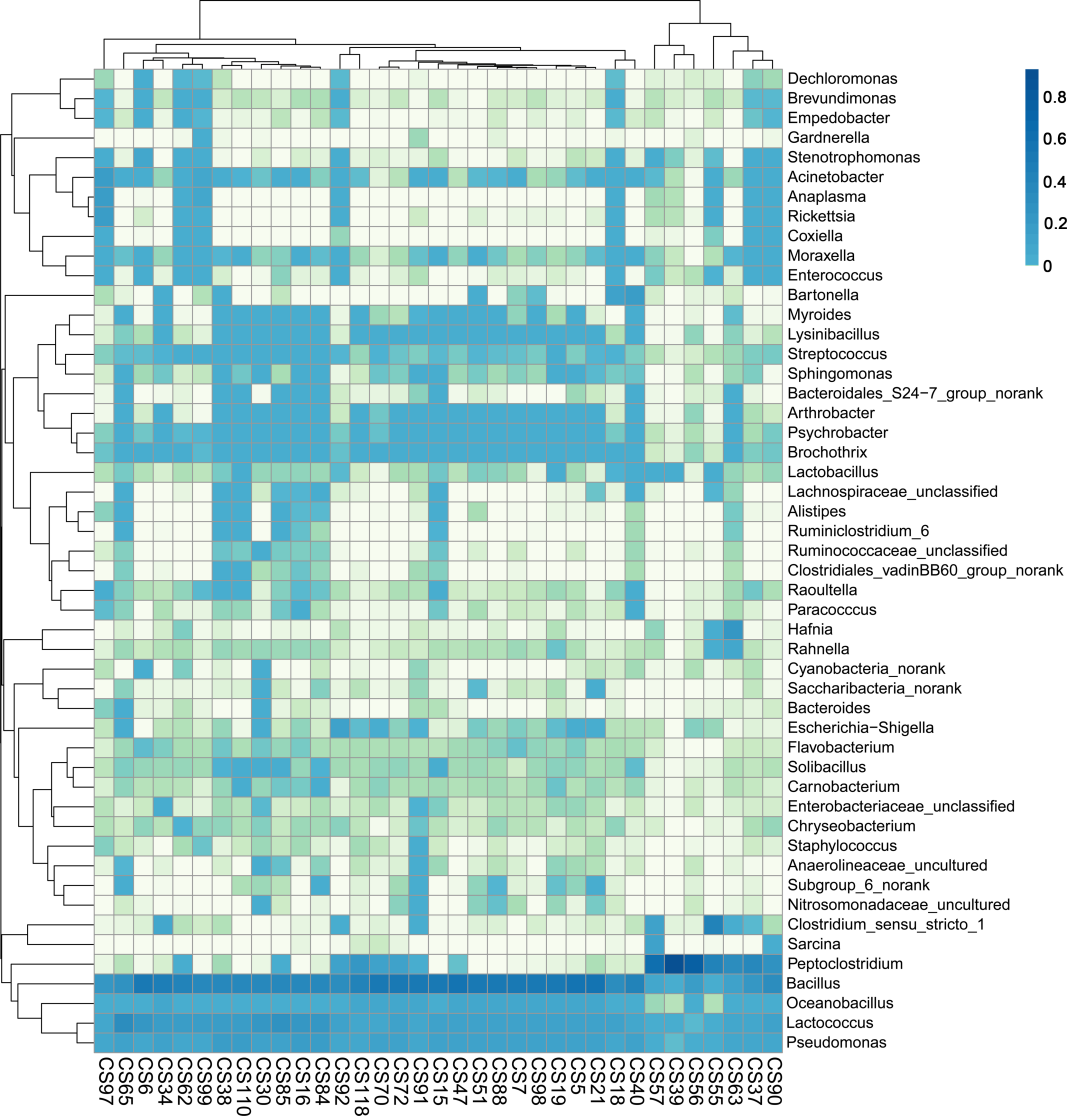
Heat map of spleen microbiota compositions at genus level. Log-scaled percentages of the abundances for the top 50 taxa at genus level in 35 mouse and shrew samples from Chongming Island, Shanghai city were illustrated in the heat map. The color scale (log10%) was on the left.

The 35 samples were hierarchically clustered against the genera with top 50 relative abundances including *Anaplasma*, *Rickettsia*, *Coxiella* and *Bartonella* but not *Ehrlichia* (Fig 3). Notably, *Anaplasma*, *Rickettsia* and *Coxiella* were adjacently clustered to each other, whereas *Bartonella* were not adjacent to these three genera. This further reflected the closely related occurrences of *Anaplasma*, *Rickettsia* and *Coxiella* but not *Bartonella* in the wild mice and shrews.

### Diversities in the spleen microbiota

**Fig 4:**
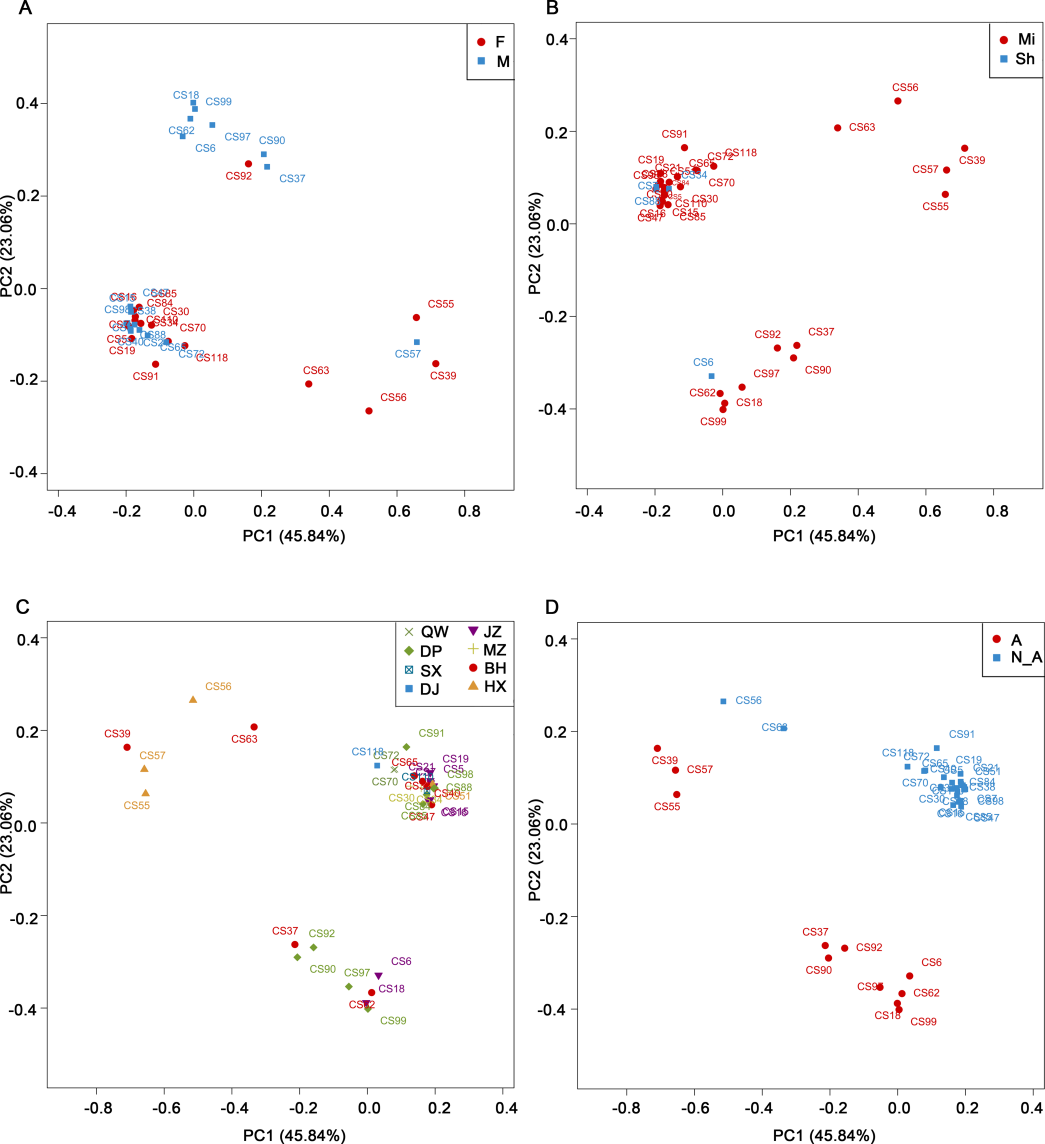
Diversities in the spleen microbiota of 35 mice and shrews. Principal coordinate analysis (PCoA) plots of the spleen microbiota based on the Bray-Curtis metrics considering animal genders, types, geographic sites or infection with *Anaplasma*. (A), male versus female groups. (B), mouse versus shrew groups. (C), different geographic site groups. (D), *Anaplasma*-positive versus *Anaplasma*-negative groups. F: female; M: male. Mi: mice; Sh: shrews. A: *Anaplasma*-positive; N_A: *Anaplasma*-negative.

Principal coordinate analyses (PCoA) based on the Bray-Curtis metrics (S2 Table) were performed to look at the overall differences in the spleen microbiota of the 35 samples considering the factors of animal genders, types, locations or infection with *Anaplasma*. *Anaplasma* had a relatively high mean abundance among the tick-borne bacteria detected in the present study. As shown in Fig 4A, two relatively dense groups and one relatively scattered group were observed and circled. One of the two relatively dense groups consisted of 22 samples including 10 male and 12 female animals (Fig 4A). Three of the 22 samples were shrews (Fig 4B). And the geographic sites of these 22 samples covered all the 8 sites in the present study (Fig 4C). All of the 22 samples were *Anaplasma*-negative (Fig 4D). The other relatively dense group consisted of 8 samples including 7 male and 1 female animals (Fig 4A). One of these 8 samples was shrew (Fig 4B). The geographic sites of the 8 samples were from the 3 sites, JZ, BH and DP (Fig 4C). And all of the 8 samples were *Anaplasma*-positive (Fig 4D). The 5 samples from the relatively scattered group were all mice and from the 2 sites, BH and HX (Fig 4B and 4C). Three of these 5 samples, CS39, CS55 and CS57, were close to each other, and they were all *Anaplasma*-positive (Fig 4D). One of these three samples was male, and 2 were female (Fig 4A). The remaining 2 samples from the relatively scattered group were more scattered, which were female animals and *Anaplasma*-negative (Fig 4A and 4D). Compared with the samples in the other relatively dense groups, these 5 samples in the relatively scattered group had exceptionally high percentages of *Peptoclostridium* and low percentages of *Bacillus* and *Lactococcus* (Fig 2), which contributed greatly to their straying away from the other two groups in the PCoA plots (Fig 4).

**Fig 5:**
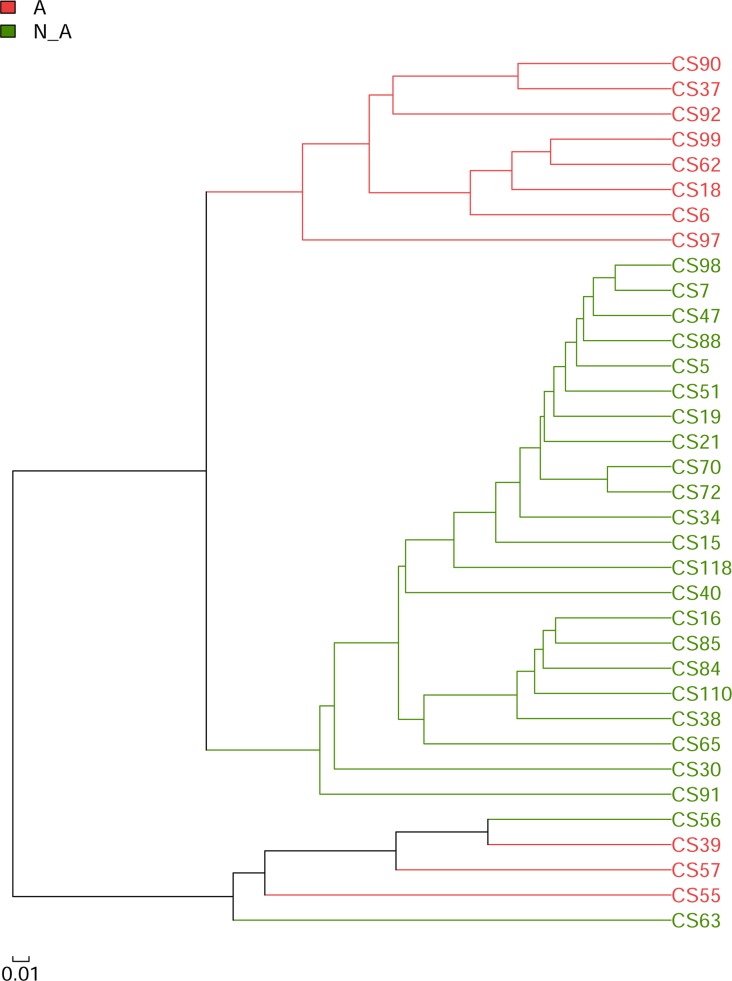
Hierarchical cluster tree. *Anaplasma*-positive and *Anaplasma*-negative samples were hierarchically clustered using UPGMA algorithm based on Bray-Curtis distances. A: *Anaplasma*-positive, in red; N_A: *Anaplasma*-negative, in green.

To further analyze the microbiota considering the factor of infection with *Anaplasma*, a hierarchical clustering using unweighted pair group method with arithmetic mean (UPGMA) was conducted to compare the microbiota similarities between *Anaplasma-*positive and *Anaplasma-*negative samples. The overall 35 samples were divided into two major clusters as shown in Fig 5. Eight of the 11 *Anaplasma-*positive samples were in the bigger cluster, and they were sub-clustered into an independent group. The other 3 of the 11 *Anaplasma-*positive samples were in the smaller cluster, and they were clustered in a consecutively order. Among the spleen microbiota of tested samples, the overall similarities indicated by hierarchical clustering (Fig 5) was consistent with the diversities revealed by the PCoA plot (Fig 4D).

### Microbiota differences between *Anaplasma-*positive and *Anaplasma-*negative samples

**Fig 6:**
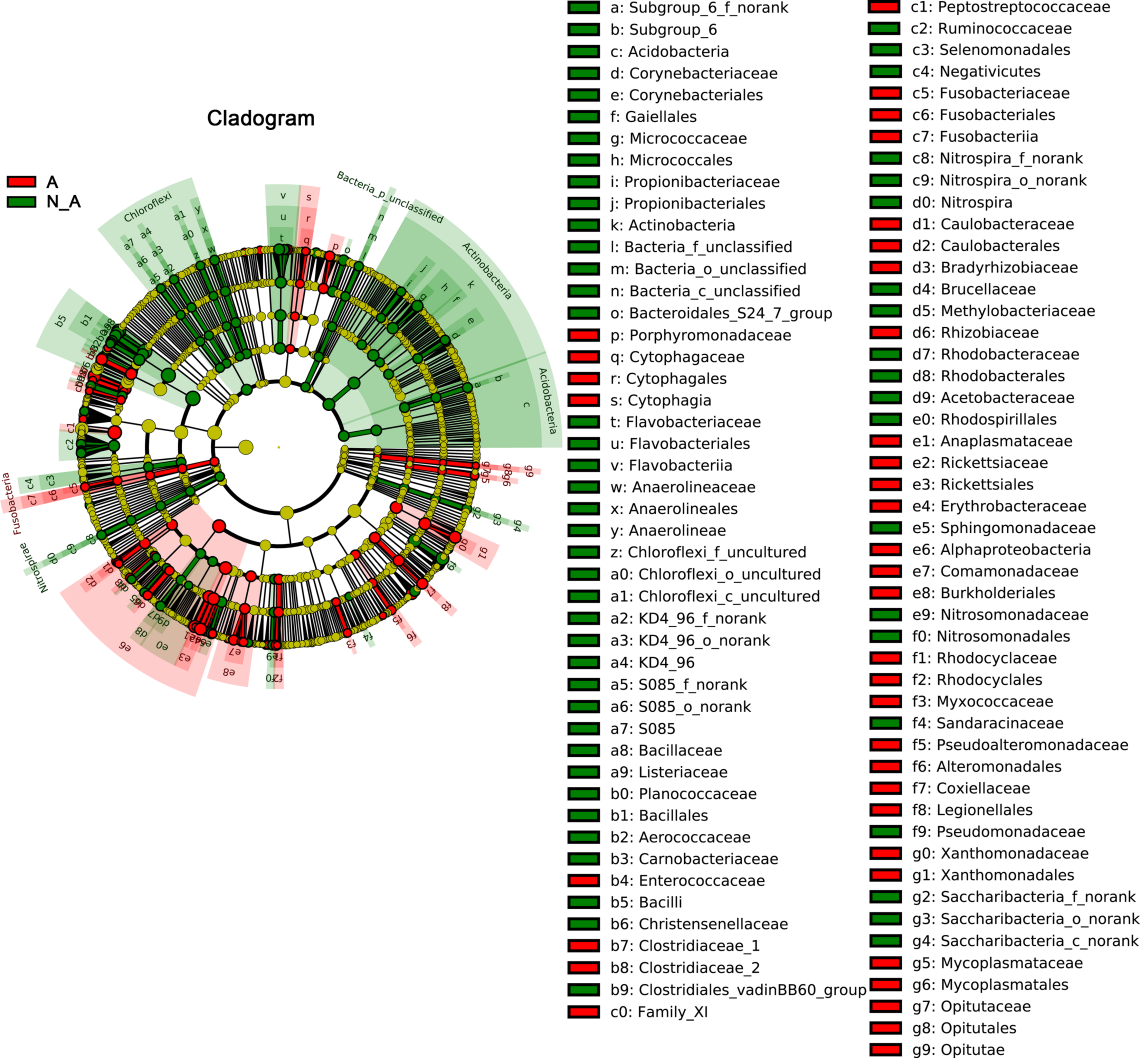
Differentially abundant bacterial taxa in the spleen microbiota between *Anaplasma*-positive and *Anaplasma*-negative samples identified by LEfSe analysis. Cladogram showed the highly represented taxa in *Anaplasma*-positive and *Anaplasma*-negative samples, respectively. A: *Anaplasma*-positive, in red; N_A: *Anaplasma*-negative, in green.

A number of differentially abundant bacterial taxa between *Anaplasma-*positive and *Anaplasma-*negative samples were identified in the spleen microbiota by the linear discriminant analysis effect size (LEfSe) analysis as shown in Fig 6. The differentially enriched taxa in *Anaplasma*-positive samples were mainly from the phyla of Proteobacteria and Fusobacteria, whereas the differentially enriched taxa in *Anaplasma*-negative samples were mainly from the phyla of Actinobacteria, Acidobacteria, Choloroflexi, Nitrospirae and Proteobacteria. Although there was no significant difference in the abundance of overall Proteobacteria phylum, there were significant differences in the class α-Proteobacteria and in some genera from the classes of β and γ- Proteobacteria between *Anaplasma-*positive and *Anaplasma-*negative samples.

**Fig 7:**
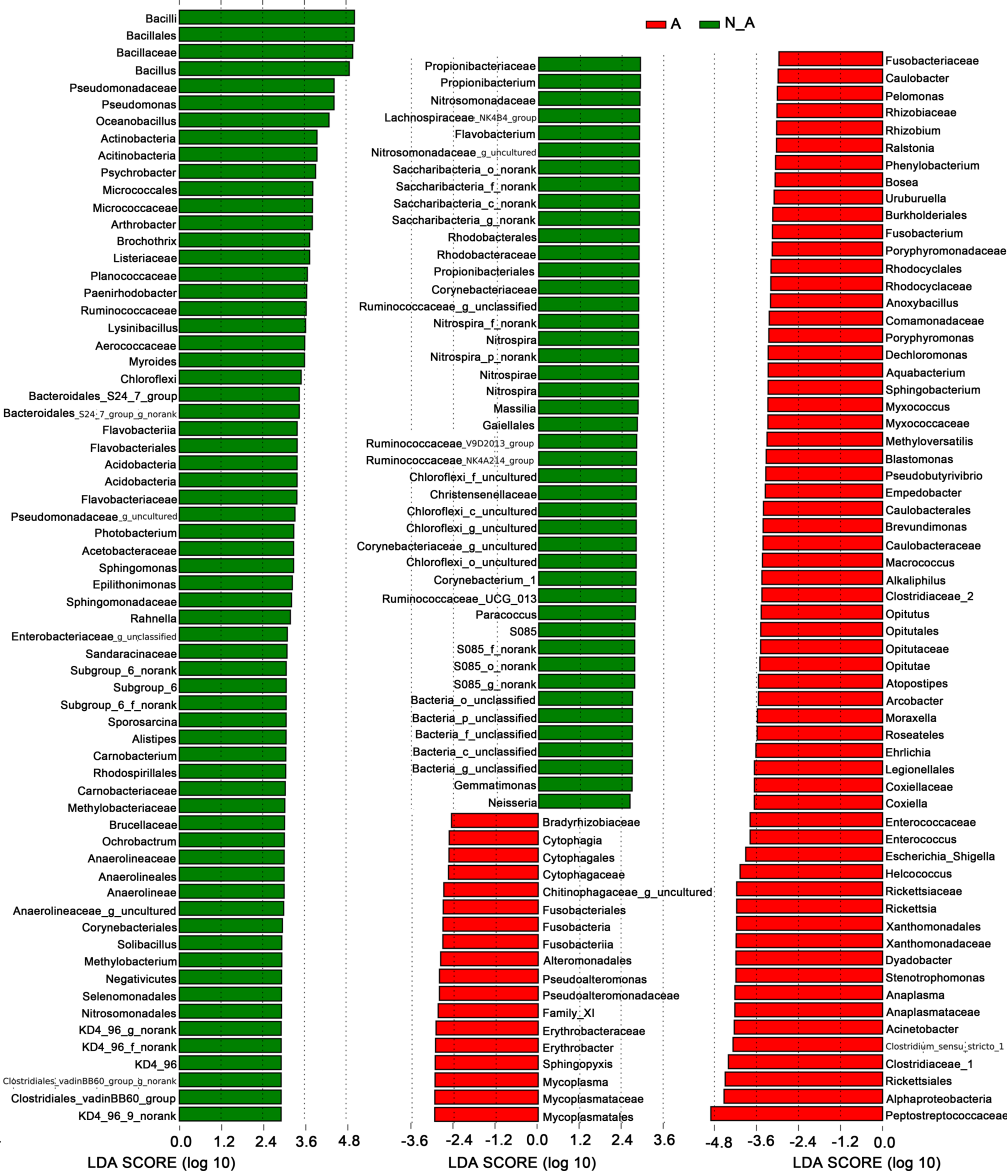
Histogram of the linear discriminant analysis (LDA) scores. Differentially abundant bacterial features enriched in *Anaplasma*-positive and *Anaplasma*-negative samples, respectively. The absolute values of LDA scores were > 2 (p < 0.05). A: *Anaplasma*-positive, in red; N_A: *Anaplasma*-negative, in green.

A comparison of the spleen microbiota between *Anaplasma-*positive and *Anaplasma-*negative samples to genus level revealed a list of differentially abundant bacterial features with absolute linear discriminant analysis (LDA) scores > 2 (Fig 7), suggesting that the *Anaplasma*-infection was associated with specific patterns of spleen microbiota from mice and shrews. The features with top 25 absolute LDA scores in *Anaplasma-*positive samples were Peptostreptococcaceae, Alphaproteobacteria, Rickettsiales, Clostridiaceae_1, *Clostridium_sensu_stricto_1*, *Acinetobacter*, Anaplasmataceae, *Anaplasma*, *Stenotrophomonas*, *Dyadobacter*, Xanthomonadaceae, Xanthomonadales, *Rickettsia*, Rickettsiae, *Helcococcus*, *Escherichia_Shigella*, *Enterococcus*, Enterococcaceae, *Coxiella*, Coxiellaceae, Leginellales, *Ehrlichia*, Roseateles, *Moraxella* and *Arcobacter* (Fig 7). The tick-borne bacteria, *Anaplasma*, *Rickettsia*, *Coxiella* and *Ehrlichia*, were all recognized within the top 25 differentially represented features of *Anaplasma-*positive samples. In contrast, the features with top 25 absolute LDA scores in *Anaplasma-*negative samples were Bacilli, Bacillales, Bacillaceae, *Bacillus*, Pseudomonodaceae, *Pseudomonoas*, *Oceanobacillus*, Actinobacteria, Actinobacteria, Psychrobacter, Micrococcales, Micrococcaceae, *Arthrobacter*, *Brochothrix*, Listericeae, Planococcaceae, *Paenirhodobacter*, Ruminococcaceae, *Lysinibacillus*, Aerococcaceae, *Myroides*, Chloroflexi, Bacteroidales_S24_7_group, Bacteroidales_S24_7_group_g_norank and Flavobacteriia. *Bartonella* were neither included in the differentially abundant bacterial taxa of *Anaplasma-*positive samples nor in those of *Anaplasma-*negative samples (Fig 7).

In addition, a few differentially abundant taxa in the spleen microbiota between male and female animals, mice and shrews or animals from multiple geographic sites at the ranks below phylum were observed by LEfSe analysis, respectively (S1 Fig). Nevertheless, no vector-borne bacteria, *Anaplasma*, *Ehrlichia*, *Rickettsia*, *Coxiella* or *Bartonella*, were identified among these differentially enriched taxa.

**Fig 8:**
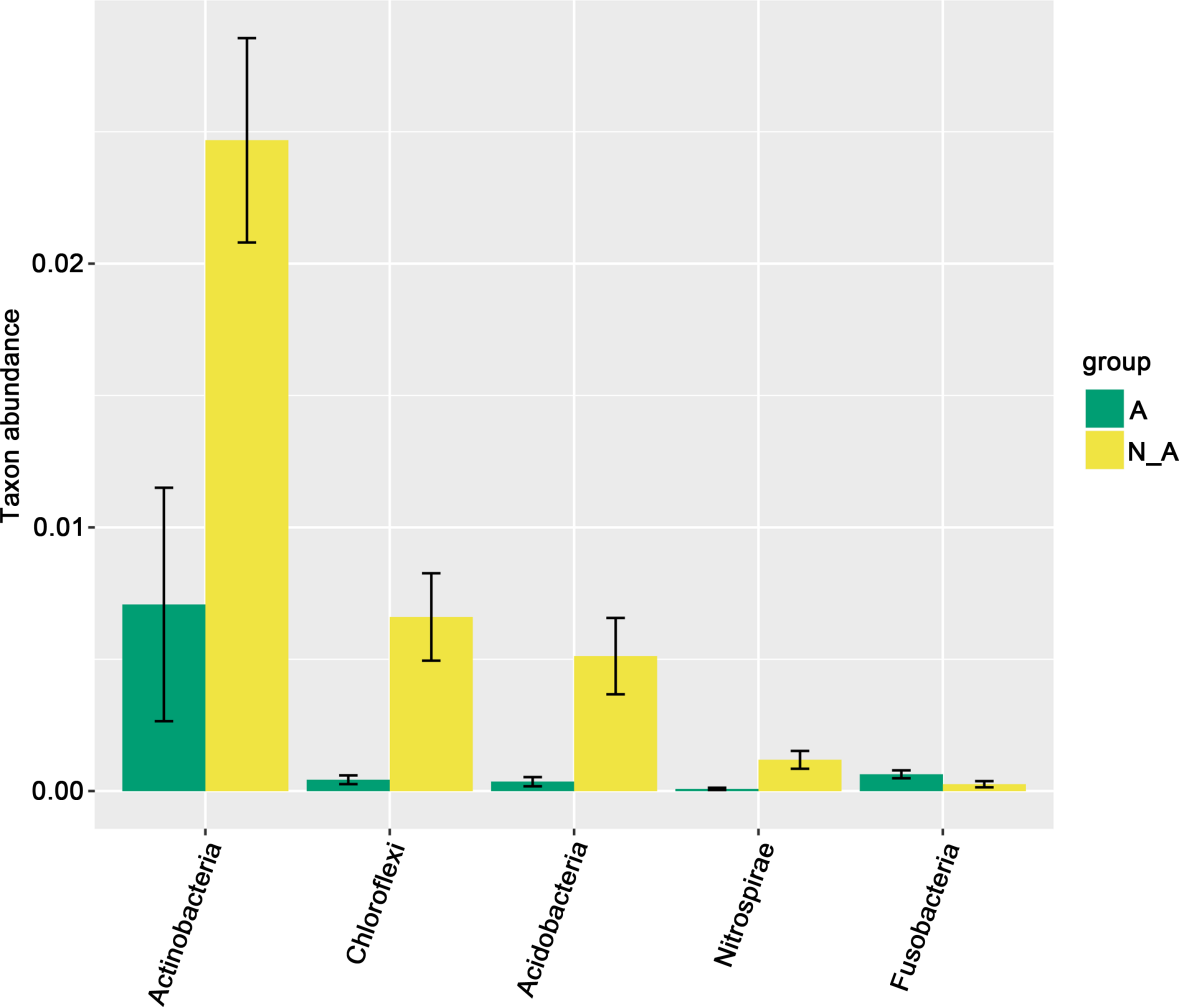
Wilcoxon rank-sum test. Differentially abundant phyla enriched in *Anaplasma*-positive and *Anaplasma*-negative samples, respectively, analyzed by Wilcoxon rank-sum test (p < 0.05; q< 0.01). A: *Anaplasma*-positive, in green; N_A: *Anaplasma*-negative, in yellow.

Wilcoxon rank-sum test was used to further compare the relative abundances of taxa between *Anaplasma-*positive and *Anaplasma-*negative samples at phylum level. Consistent with LEfSe analysis (Fig 7), Actinobacteria, Chloroflexi, Acidobacteria and Nitrospirae were significantly more abundant in *Anaplasma-*negative samples than in *Anaplasma-*positive samples, whereas Fusobacteria was significantly more abundant in *Anaplasma-*positive samples than in *Anaplasma-*negative samples based on the analysis of Wilcoxon rank-sum test (Fig 8). At genus level, 44 significantly different genera were identified between *Anaplasma-*positive and *Anaplasma-*negative samples (S2 Fig) by the analysis of Wilcoxon rank-sum test, which was consistent with the result from LEfSe analysis too (Fig 7). *Anaplasma*, *Rickettsia*, *Coxiella* and *Ehrlichia* were all significantly more abundant in *Anaplasma-*positive samples than in *Anaplasma-*negative samples by the analysis of Wilcoxon rank-sum test (S2 Fig).

## Discussion

The present study has comprehensively characterized the spleen microbiota in wild mice and shrews from Chongming Island, Shanghai City, which has advanced our understanding on spleen microbiota in wild animals. To our knowledge, this is the first time that bacterial profiles in the spleens of wild animals have been explored in China. It has provided a wealth for the comparative analyses of spleen microbiota across different types of mammalian hosts from different geographic areas. The present study is the first report on unique bacterial taxa associated with tick-borne bacteria in wild mammals. This study has further proved the application of 16S rDNA metagenomics as a powerful methodology to study the prevalence of bacteria in the circulatory system of wild life as suggested by *Razzauti et al*. [32].

### Microbiota of blood and the spleen

In the present study, the two major bacterial phyla among the taxa of tested mouse and shrew samples were Firmicutes and Proteobacteria. Previous reports have shown that microbiota of blood and the spleen from humans or mice were mainly consisted of Proteobacteria and sometimes Firmicutes as well. For instance, the major prevalent bacterial genus in both gerbil rodent blood samples from Israel and mouse spleen samples from France was *Bartonella*, belonging to the phylum Proteobacteria [31,32]. Firmicutes and Proteobacteria were the two major phyla with comparable relative abundances in the blood microbiota of healthy human samples [26]. The predominant phylum, Proteobacteria, represented 90% of the overall microbiota in human blood samples [35]. Greater than 80% of the blood microbiota in 30 healthy blood donors was from the phylum Proteobacteria followed by the phyla of Actinobacteria, Firmicutes and Bacteroidetes [25]. The blood microbiota in nonalcoholic fatty liver disease (NAFLD) patients mainly consisted of Proteobacteria (87.9%), which was followed by Actinobacteria (7.3%), Firmicutes (3.7%) and Bacteroidetes (1.1%) [36]. Although there was variation in the proportions of Proteobacteria and Firmicutes between the spleen microbiota of wild mice and shrews in the present study and the blood and spleen microbiota of humans or mice from the aforementioned reports, the overall blood or spleen microbiota in humans or mice is different from the gut microbiota, which is dominated by Firmicutes and Bacteroidetes [37].

Intriguingly, the present study has shown that the infection of wild mice and shrews with *Anaplasma* has been associated with a specific spleen microbiota. A number of significantly differentially abundant bacterial taxa between *Anaplasma-*positive and *Anaplasma-*negative samples were revealed by both LEfSe analysis and Wilcoxon rank-sum test, respectively. As shown in Fig 5, the 35 tested samples fell into two major clusters based on the analysis using unweighted pair group method with UPGMA. Eight *Anaplasma*-positive samples were independently sub-clustered within the bigger major cluster, and 3 *Anaplasma*-positive samples were adjacently clustered within the smaller major cluster. It seemed that the formation of these two major clusters were resulted from some unknown factors, which were different from the factors analyzed in the present study i.e., animal genders, types, geographic sites or infection with *Anaplasma* (Fig 4). Additionally, it was interesting to notice that there were a few differentially enriched taxa identified in the spleen microbiota from animals with different genders, types or geographic locations at the rank levels below phylum by LEfSe analysis in the present study, suggesting that the factors of animal genders, types or geographic locations had impacts on the spleen microbiota.

### Vector-borne bacteria

The present study is the first report on the detection of vector-borne bacteria, *Anaplasma*, *Ehrlichia*, *Rickettsia*, *Coxiella* and *Bartonella*, in Chongming Island, which suggests that the wild mice and shrews serve as important animal reservoirs for these vector-borne bacteria in the studied areas. *Anaplasma*, *Ehrlichia*, *Rickettsia*, *Coxiella* and *Bartonella* were detected from multiple foci in Chongming Island in the present study, which reflected their relative wide distributions in this island. Among these bacteria, only *Ehrlichia* was not detected in shrews, which was probably due to the small quantities of shrew samples in the present study. Eight of 11 *Anaplasma* were male animals, which could be resulted from differences in the ecological behaviors between male and female wild mice and shrews tested in this study. This was less likely due to any potentially intrinsic differences in the spleen niche between male and female animals since *Anaplasma*, *Ehrlichia*, *Rickettsia*, *Coxiella* or *Bartonella* were not among the differentially enriched taxa identified in the spleen microbiota of animals with different genders. Furthermore, these bacteria were not among the differentially enriched taxa in the spleen microbiota of mouse versus shrew groups or different geographic site groups either, suggesting that infection with these bacteria was neither specific to mice or shrew nor to a single geographic site in Chongming Island.

The closely related occurrences of *Anaplasma*, *Ehrlichia*, *Rickettsia* and *Coxiella* in the present study suggested that *Anaplasma*, *Ehrlichia*, *Rickettsia* and *Coxiella* shared the transmission routes in the studied areas. Both of *Ehrlichia* and *Anaplasma* belong to the family Anaplasmaceae and are tick-borne bacteria. Many members in the SFG *Rickettsia* are transmitted by ticks. *Coxiella* can be transmitted by ticks too. It was very likely that *Anaplasma*, *Ehrlichia*, *Rickettsia* and *Coxiella* in the co-infected animals in the present study were transmitted by ticks instead of other vectors. Therefore, there was a high chance to get infected with multiple of them upon a tick exposure. *A*. *ovis* was the major prevalent *Anaplasma* sp. in the present study. *A*. *ovis* infects ruminants and causes ovine anaplasmosis. *A*. *phagocytophilum*, a zoonotic pathogen, can cause anaplasmosis in both humans and animals. Infection with *Ehrlichia* causes febrile diseases in mammalian hosts. *Coxiella* and *Rickettsia* were usually considered as vector-borne pathogens. However, with the advance of molecular biology, some members of *Coxiella* and *Rickettsia* are gradually recognized as non-pathogenic intracellular bacteria, which are actually endosymbionts to their hosts [38].

*Coxiella*_endosymbiont_of_*Rhipicephalus*_*turanicus*, also called *Coxiella*-like endosymbiont (*Coxiella*-LE), was the only member of *Coxiella* detected in this study. Coxiella-LE distributes in ticks worldwide [38]. *Coxiella* and *Rickettsia* were among the ten maternally inherited bacteria found in ticks summarized by Bonnet, i.e., *Coxiella*-LE, *Rickettsiella*, *Arsenophonus*, *Francisella*-LE, *Cardinium*, *Spiroplasma*, *Lariskella*, *Midichloria*, *Rickettsia* and *Wolbachia* [38]. Besides *Coxiella* and *Rickettsia*, *Rickettsiella* were detected in our study, too. *Rickettsiella* was transferred from the order Rickettsiales to the family Coxiellaceae in the order Legionellales based on the phylogenetic analysis of 16S rRNA sequences [39]. Nevertheless, *Rickettsiella* were only detected in sample CS57 and had a much less relative abundance in the present study. There may be new tick borne-bacteria present in the differentially abundant bacterial taxa of *Anaplasma-*positive samples revealed in the present study.

In the present study, the infection rate of *Rickettsia* was 60%, which was the highest among the vector-borne bacteria detected. The *Rickettsia-*positive samples covered all *Anaplasma*, *Ehrlichia* or *Coxiella*-positive samples but not all *Bartonella*-positive samples. *Rickettsia* was prevalent in mice and shrews from all types of environment investigated in the present study, i.e., forests, agricultural fields, residential areas and banks. In contrast, *Anaplasma*, *Ehrlichia* or *Coxiella* were detected in mice and shrews from the forests and agricultural fields rather than residential areas or banks. Compared with residential areas and banks, the forests and agricultural fields in the studied areas had more green coverage and were more suitable for tick survival. It was unclear whether the *Rickettsia* spp. from the animals co-infected with *Anaplasma*, *Ehrlichia* or *Coxiella* in the forests and agricultural fields were same as those prevalent in residential areas and banks in the present study. Furthermore, it was unclear whether the vectors transmitting *Anaplasma*, *Ehrlichia*, *Coxiella* or *Rickettsia* in the forests and agricultural fields were same as those transmitting *Rickettsia* in residential areas and banks in this study either.

*Bartonella* in the present study were probably transmitted by vectors other than ticks. *Bartonella* can be transmitted by several arthropod vectors such as fleas, keds, lice, sand flies and ticks, or direct bites by infected animals and often establish persistent infection in asymptomatic mammalian hosts [40]. *Bartonella* were the most frequently identified bacteria in the fleas collected from southern Indiana, USA [41]. *Bartonella* together with *Mycoplasma* were the dominant flea-borne bacteria detected in gerbil rodent blood samples from Israel [31]. Haemotrophic *Mycoplasma* has different subgroups and been detected in the spleen or blood samples of rodents [42]. In the present study, however, the relative abundance of *Mycoplasma* was less than 1%. Both *Bartonella* and *Mycoplasma* were among the 23 features detected in the rodent blood and/or flea samples summarized by Cohen et. al. [31]. Besides *Bartonella*, *Mycoplasma* and *Rickettsia*, other 14 of these 23 features i.e., *Aquabacterium*, *Bifidobacterium*, *Bradyrhizobium*, Cyanobacterium (phylum), *Halomonas*, *Lactobacillus*, *Massilia*, *Methylobacterium*, *Neisseria*, *Ralstonia*, Rhizobiales_unclassified (order), *Staphylococcus*, *Streptococcus* and Sphingobacteria (class), were detected in the present study too. The remaining 6 of the 23 features, *Azovibrio*, *Catenuloplanes*, *Diaphorobacter*, *Saccharothrix*, *Spirosoma* and *Wolbachia*, were not detected in the present study.

*Bartonella* were also the most prevalent genus in vector-borne bacteria detected in the spleen microbiota of wild voles from France [32]. There were 45 potential zoonotic bacterial genera in total detected by Razzauti M *et al* [32]. Eleven of the 45 genera, *Anaplasma*, *Bacillus*, *Bartonella*, *Clostridium*, *Coxiella*, *Escherichia/ Shigella*, *Moraxella*, *Rickettsia*, *Staphylococcus*, *Stenotrophomonas* and *Streptococcus*, were among the relatively abundant bacterial genera listed in Fig 2 in the present study. Twenty one of the 45 genera, *Aeromonas*, *Burkholderia*, *Campylobacter*, *Corynebacterium*, *Ehrlichia*, *Enterococcus*, *Eubacterium*, *Granulicatella*, *Haemophilus*, *Helicobacter*, *Leptospira*, *Mannheimia*, *Micrococcus*, *Mycobacterium*, *Mycoplasma*, *Neisseria*, *Neochlamydia*, *Pasteurella*, *Rhodococcus*, *Treponema* and *Vibrio*, were detected with relatively low abundance in the present study (S1 Table) and hence not listed in Fig 2. The left 13 genera, *Bordetella*, *Borrelia*, *Brucella*, *Francisella*, *Klebsiella*, *Legionella*, *Listeria*, *Orientia*, *Salmonella*, *Ureaplasma* and *Yersinia* were not detected in the present study. The prevalence of *Rhodococcus*, *Legionella*, *Staphylococcus*, *Corynebacterium*, *Streptococcus* and *Stenotrophomonas*, reported to contaminate laboratory reagents [43], were high in the spleen microbiota of wild voles from France, and the authors suspected that the presence of bacteria in the samples were due to contamination instead of real infection of the animals [32]. In the present study, however, *Rhodococcus* and *Corynebacterium* were the genera with relatively low abundance, and *Legionella* was not detected. And *Staphylococcus*, *Stenotrophomonas* and *Streptococcus* were the 37^th^, 14^th^ and 20^th^ abundant genera in the present study, respectively. Blank controls were set throughout the 16S metagenomics sequencing in our study, and the detection of these bacteria was very likely due to real infection of wild animals rather than contamination of samples. However, as emphasized by *Razzauti et al*. [32], caution should be taken when DNA-based techniques are used to detect microbes.

In future, it would be important to investigate the molecular characteristics of vector-borne bacteria prevalent in the studied areas. At the same time, it would be also important to characterize the vectors. These will contribute to the prevention and control of vector-borne bacterial infection in the studied areas. Furthermore, it would be interesting to study the interaction between tick-borne bacteria and their host cells in the spleen. Studies on the mechanism underlying the alteration of the spleen microbiota due to infection with tick-borne bacteria would not only advance the knowledge of the pathogenesis of tick-borne bacteria but also shed light on the function of spleen microbiota from the perspective of infection.

## Supporting information

S1 Fig Histograms of the linear discriminant analysis (LDA) scores. Differentially abundant genera enriched in males versus females (A), in mice versus shrews (B) and in animals from different sites (C), respectively. C, Geographic site groups with less than 3 samples, MZ, QW, SX and DJ, were not included. The absolute values of LDA scores were > 2 (p < 0.05). F: female; M: male. Mi: mice; Sh: shrews.
(TIF)

S2 Fig Wilcoxon rank-sum test. Differentially abundant genera enriched in *Anaplasma*-positive and *Anaplasma*-negative samples, respectively, analyzed by Wilcoxon rank-sum test (p < 0.05; q< 0.01). A: *Anaplasma*-positive, in green; N_A: *Anaplasma*-negative, in yellow.
(TIF)

S1 Table Spleen microbiota compositions to species level in the 35 spleen samples of small wild mammals from Chongming Island, Shanghai city. (XLS)

S2 Table Bray-Curtis metrics of the spleen microbiota of 35 small wild mammals from Chongming Island, Shanghai city. (XLSX)
