# Supplementary figures and images for: The spleen microbiota of small wild mammals reveals distinct patterns with tick-borne bacteria
Source: PLoS Negl Trop Dis. 2018 Jul 5;12(7):e0006499. doi: 10.1371/journal.pntd.0006499 (PMC6033388; doi:10.1371/journal.pntd.0006499)

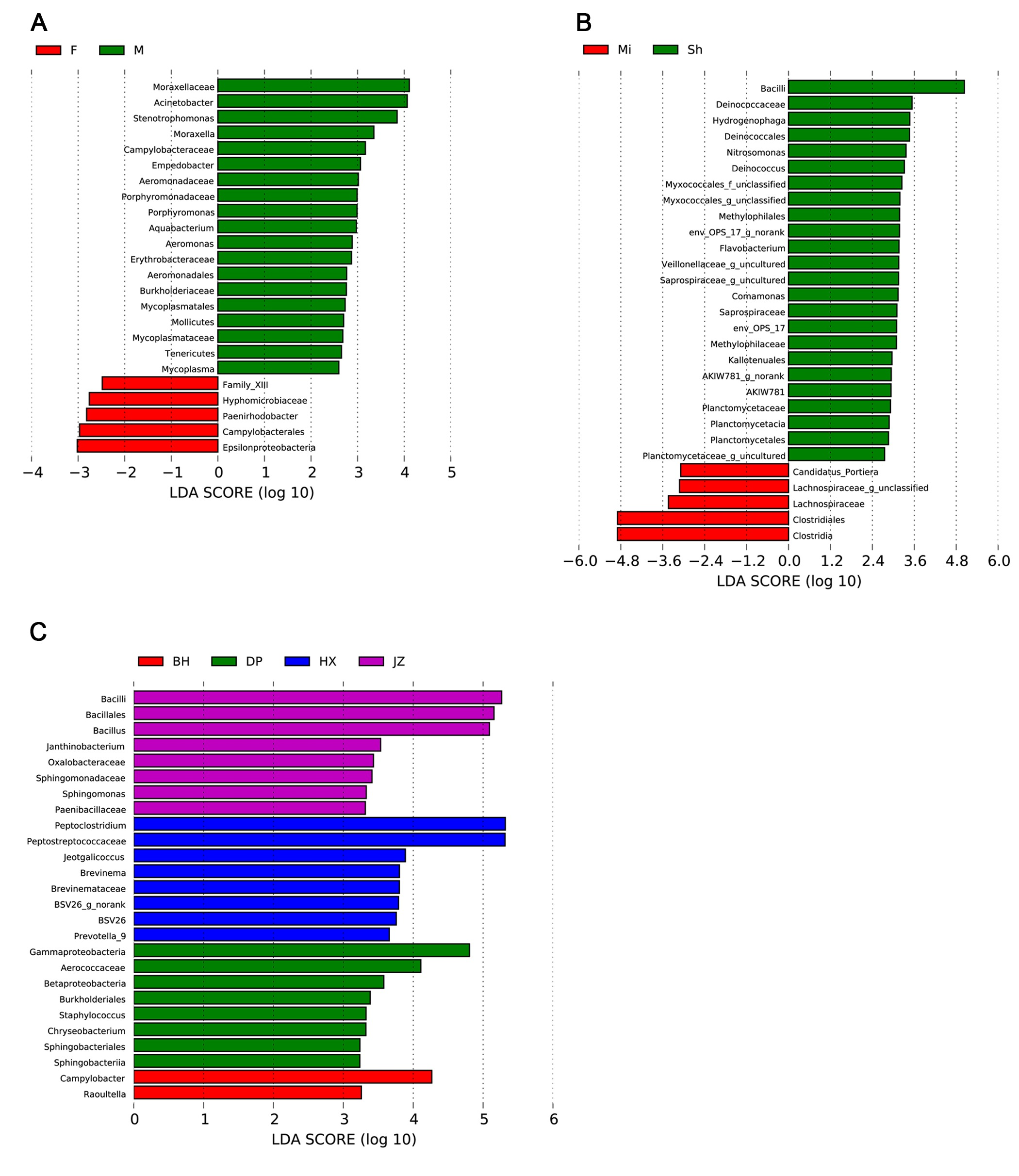

Supplement: S1 Fig — Differentially abundant genera enriched in males versus females (A), in mice versus shrews (B) and in animals from different sites (C), respectively. C, Geographic site groups with less than 3 samples, MZ, QW, SX and DJ, were not included. The absolute values of LDA scores were > 2 (p < 0.05). F: female; M: male. Mi: mice; Sh: shrews. (TIF) [file pntd.0006499.s001.tif]

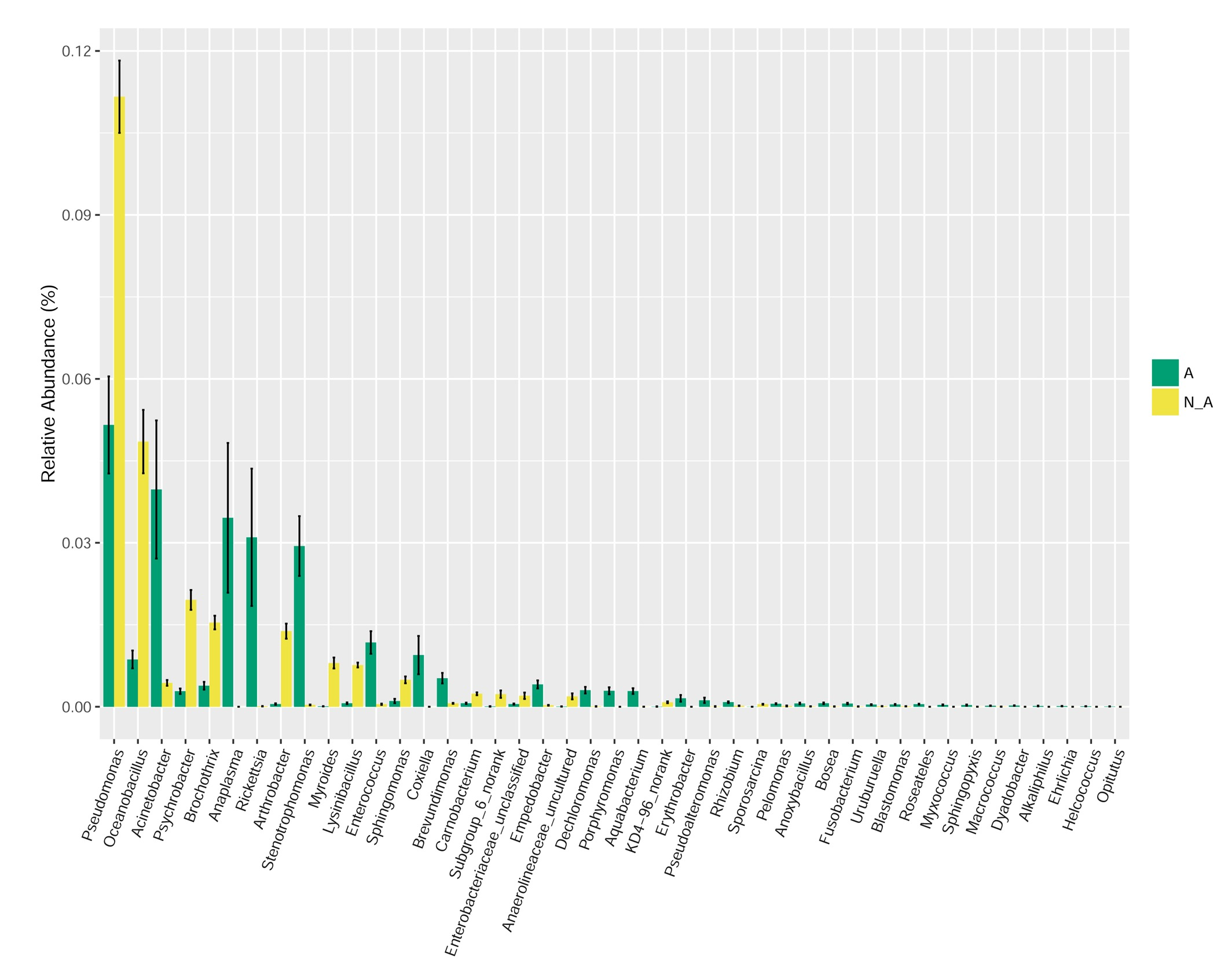

Supplement: S2 Fig — Differentially abundant genera enriched in Anaplasma-positive and Anaplasma-negative samples, respectively, analyzed by Wilcoxon rank-sum test (p < 0.05; q< 0.01). A: Anaplasma-positive, in green; N_A: Anaplasma-negative, in yellow. (TIF) [file pntd.0006499.s002.tif]
